# Antioxidant and Angiostatic Effect of *Spirulina platensis* Suspension in Complete Freund’s Adjuvant-Induced Arthritis in Rats

**DOI:** 10.1371/journal.pone.0121523

**Published:** 2015-04-08

**Authors:** Eman A. I. Ali, Bassant M. Barakat, Ranya Hassan

**Affiliations:** 1 Department of Histology & Cell Biology, Faculty of Medicine, Suez Canal University, Ismailia, Egypt; 2 Department of Pharmacology & Toxicology, Faculty of Pharmacy (Girls), Al Azhar University, Cairo, Egypt; 3 Department of Clinical Pathology, Faculty of Medicine, Suez Canal University, Ismailia, Egypt; Indian Institute of Integrative Medicine, INDIA

## Abstract

**Background:**

Currently, natural products have built a well-recognized role in the management of many degenerative diseases, mainly rheumatoid arthritis. Recent studies suggest that Spirulina, a unicellular blue-green alga, may have a variety of health benefits and curative properties and is also competent of acting as an anti-inflammatory, antioxidant and recently anti-angiogenic agent. In the present study, the antioxidant and the immunomodulatory effect of *Spirulina platensis* as well as its anti-angiogenic effect against complete Freund's adjuvant-induced arthritis (AIA) in rat model were tested.

**Results:**

We found that the development of arthritis was concealed; moreover it successfully inhibited the development of macroscopic as well as microscopic and histopathological lesions in AIA rats when compared to control. Spirulina treated group showed a higher survival rate and moreover, it reduced the clinical score of RA in a dose dependent manner. Furthermore, Spirulina decreased serum levels of COX-2, TNF-α, IL-6, TBARS, VEGF and increased serum levels of GSH compared to the RA non-treated group.

**Conclusions:**

The present study concluded that Spirulina is able to restrain the changes produced through adjuvant-induced arthritis. The suppressing effect of Spirulina could be attributed, at least in part, to anti-inflammatory, antioxidant and anti-angiogenic properties.

## Introduction

Rheumatoid arthritis (RA) is an inflammatory progressive, disabling autoimmune disease [1] characterized by systemic inflammation, persistent synovitis, and production of autoantibodies [2,3]. RA has many complications e.g. joint damage, disability, decreased quality of life, cardiovascular and other comorbidities. It also has serious physical and emotional consequences [4,5].

The pathogenesis of RA remains obscure. Several mechanisms contribute to synovial inflammation, including T-cell activation, persistence of cytokine networks as tumor necrosis factor-α (TNF-α), interleukin-1β (IL-1β) and IL-6, antibodies to synovial tissue, proinflammatory small molecules and angiogenesis [6,7]. Angiogenesis of the inflamed synovium is a hallmark of RA [8]. It appears to result from local hypoxia, generation of reactive oxygen species (ROS) and subsequent lipid peroxidation and growth factor production like fibroblast growth factors (FGF-1 and FGF-2), vascular endothelial growth factor (VEGF), IL-1, IL-8 and TNF-α. All these factors induce production, migration, and differentiation of macrophages, synovial cells, and endothelial cells [9, 10, 11].

There are three general modules of drugs used in the treatment of RA: non-steroidal anti-inflammatory agents (NSAIDs), corticosteroids, and disease modifying anti-rheumatic drugs (DMARDs) [12]. NSAIDs and corticosteroids have a shorter onset of action than DMARDs [13]. The treatment goals of RA include achieving remission and preventing further damage and loss of function of the joints, without causing permanent side effects [14]. Targeting angiogenesis should yield new therapeutic options in the future, expanding upon already successful treatments.

Spirulina is a type of simple one-celled microscopic fresh-water blue green algae that grows naturally in warm climates and has been taken as supplement in human and animal food [15]. Spirulina is known to have a diverse biological activity due to its high content of minerals, fatty and amino acids, vitamins and it also contains phenolic acids, tocopherols and beta-carotene that are known to exhibit antioxidant properties [16]. Over the last few years, Spirulina has been found to have many additional pharmacological properties mainly through its active constituent; C-phycocyanin which exhibit anti-inflammatory, neuroprotective, immunomodulatory, anticancer activities and recently anti-angiogenic effects. Additionally, Spirulina has been reported to amend organ toxicities induced by heavy metals [17].

Taking into considerations the nutritional and pharmacological properties of Spirulina, as well as being non-toxic, bioavailable and provide considerable multiorgan protection against a lot of drugs, the present study was designed to investigate the antioxidant and angiostatic effect of Spirulina against AIA in rats in order to find an alternative safe natural remedy for treatment of RA.

## Materials and Methods

### Animals

Experiments were carried out using healthy male Wistar rats weighing 250–300 g and purchased from the Modern Veterinary Office for Laboratory Animals (Cairo, Egypt). Rats were housed in groups of four in well ventilated opaque polypropylene cages. Cage substrate was changed twice weekly with food and tap water *ad libitum*. Rats were maintained at 22±3°C under normal light and dark cycle (lights on at 4:30 a.m.). Rats were allowed to acclimatize for one week prior to use. All experimental procedures were approved by the Research Ethics Committee at the Faculty of Pharmacy, Suez Canal University (Ismailia, Egypt).

### Drugs and chemicals

Complete Freund’s adjuvant (CFA) was purchased from Sigma-Aldrich (MO, USA). Indomethacin powder was kindly provided by Medical Union Pharmaceuticals (MUP, Ismailia, Egypt) and dissolved in sterile saline. Pure premium Spirulina platensis powder was purchased from HerbaForce (UK) and suspended in distilled water for oral administration. Enzyme Linked Immunosorbent Assay (ELISA) kits for TNF-α and VEGF were purchased from Biosource International Inc. (Camarillo, CA, USA). ELISA kits for determination of IL-6 and cyclooxygenase 2 (COX 2) were supplied by Glory Science Co. Ltd (Del Rio, TX, USA). Spectrophotometric kits for malondialdehyde and reduced glutathione were purchased from Bio-diagnostic Company (Cairo, Egypt).

### Experimental design

Rats were divided into five groups, eight animals each. Group 1 served as vehicle control and received an injection of 0.25 ml of paraffin oil into the left hind paw. All the other groups received a single subcutaneous injection of 0.25 ml of CFA in the palmar surface of the left hind paw to induce experimental adjuvant-induced arthritis (AIA) [18]. CFA, containing 1 mg heat killed mycobacterium tuberculosis per ml, was stored at 4°C and was shaken well before use. Four days after induction of arthritis, rats were started on various drug regimens that continued up to four weeks. Group 2: assigned as AIA control. Group 3: received indomethacin (5 mg/kg/day, p.o.). Group 4 & 5 received Spirulina (200 or 400 mg/kg/day, p.o.), respectively.

### Evaluation of arthritis swelling

Joint swelling was scored (arthritis index) according to the standardized method by an experienced observer [19]. Briefly, a score of 0–4 was assigned as follows: 0, no evidence of hyperemia and/or inflammation; 1, hyperemia with little or no paw swelling, 2, swelling confined predominantly to the ankle region, with modest hyperemia, 3, increased paw swelling and hyperemia of the ankle and metatarsal regions, and 4, maximal paw swelling and hyperemia involving the ankle, metatarsal, and tarsal regions.

### Blood collection and serum separation

At the end of the experiment (day 32), rats were anesthetized with thiopental sodium (50 mg/kg) [20] and killed by decapitation. A midline incision was made and blood samples were withdrawn from the heart by cardiac puncture., Blood samples were processed by centrifugation at 2000 × *g* for 15 min. Then, serum samples were separated, collected in clean tubes and stored at -80°C until use.

### Determination of thiobarbituric acid reacting substances (TBARS) and reduced glutathione (GSH)

Tissue thiobarbituric acid reacting substances (TBARS) were estimated according to the spectrophotometric method of [21] using 1, 1, 3, 3-tetramethoxypropane as a standard. Concentration of total glutathione (GSH and GSSG) and oxidized glutathione (GSSG) were measured spectrophotometrically using commercial kits according to the instructions of the manufacturer. Total GSH content was expressed in μM per g protein.

### Determination of serum level of TNF-α, IL-6, COX- 2, and VEGF

Enzyme Linked Immunosorbent Assay (ELISA) kits for TNF-α (Biosource International Inc., Camarillo, CA, USA), IL-6 (Glory Science Co., Ltd, Del Rio, TX, USA), cyclooxygenase 2 (COX- 2; Glory Science Co., Ltd, Del Rio, TX, USA) and vascular endothelial growth factor (VEGF; Biosource International Inc., Camarillo, CA, USA) were used to measure these parameters in serum samples. The method was conducted according to the manufacturer’s instructions and absorbance was read at 420 nm using an automated ELISA reader (Metertech, M960).

### Hind paw processing and histopathological examination

The left hind paw joint from each rat was cut about 0.5 cm above and below the joint. All the skin and muscles were trimmed away so that the joint was left with an intact synovial membrane. The joints were decalcified by immersion in 3% nitric acid solution and maintained at room temperature for an average of 5–7 days. The acid solution was changed every 24 h, decalcification was monitored by gently feeling the consistency of the bone by the tips of the fingers until it became firm to soft. Then joints were fixed in 10% neutral buffered formalin for 2 days. The decalcified specimens were dehydrated in alcohol series, cleared in xylene and embedded in paraffin.

Sections were serially cut at 5-μm thickness, stained with hematoxylin and eosin (H&E) or Masson’s trichrome stain and evaluated microscopically using a light microscope (CX21; Olympus, Tokyo, Japan). For H&E stained ankles, cartilage and bone destruction by synovial proliferation, cellular infiltration, pannus formation and cartilage erosion in each preparation on two separate locations were evaluated blindly, using the following scoring system [22].

- *Synovial proliferation*: Grade 0, no proliferation; Grade 1, mild proliferation with two to four layers of reactive synoviocytes; Grade 2, moderate proliferation with four layers of reactive synoviocytes, increased mitotic activity and mild or absent synovial cell invasion of adjacent bone and connective tissue; and Grade 3, severe proliferation characterized by invasion and effacement of joint space and adjacent cartilage, bone and connective tissue.
- *Cellular infiltration*: Grade 0, no changes; Grade 1, few focal infiltrates; Grade 2, extensive focal infiltrates; and Grade 3, extensive infiltrates invading the capsule with aggregate formation.
- *Cartilage erosion*: Grade 0, no changes; Grade 1, superficial, localized cartilage degradation in more than one region; Grade 2, localized deep cartilage degradation; and Grade 3, extensive deep cartilage degradation at several locations.
- *Pannus formation*: Grade 0, no changes; Grade 1, pannus formation at up to two sites; Grade 2, pannus formation at up to four sites, with infiltration or flat overgrowth of joint surface; and Grade 3, pannus formation at more than four sites or extensive pannus formation at two sites. The summations of these four scores were averaged and compared for each group. For Masson’s trichrome stained sections; the color area percentage of the green color (collagen) was measured using (Image-Pro Plus 6.3) analysis program.

### Statistical analysis

All data were expressed as means±SEM and analyzed using the Statistical Package of Social Sciences (SPSS) program version 17, (Chicago, IL, USA). Comparison between data with Gaussian distribution was carried out using Kruskal Wallis Test to compare the median of each group. The difference of mean values between groups was assessed by Bonferroni’s multiple comparisons test. For histopathological analysis, statistical analysis was performed using a nonparametric test. Mortality data was analyzed using Fisher's Exact Test. All *P* values reported are two-tailed and *P* <0.05 was considered significant.

## Results

**Fig 1:**
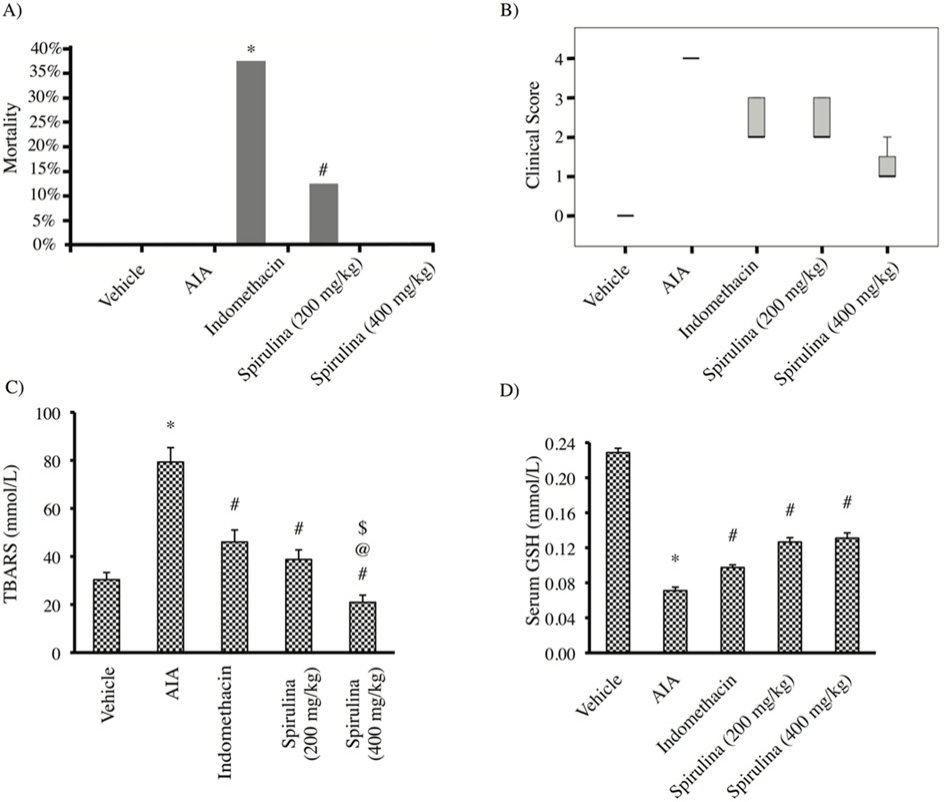
Mortality in the experimental groups by Fisher's Exact Test, * p value = 0.2 # p value = 1.0(a). (b) Effect of Spirulina (200 or 400 mg/kg) vs. indomethacin on the clinical score of the ankles, P<0.001 (Kruskal Wallis Test was statistically significant at 95% confidence level). (c) Effect of Spirulina (200 or 400 mg/kg) vs. indomethacin on the serum TBARS level. (d) Effect of Spirulina (200 or 400 mg/kg) vs. indomethacin on serum GSH level. *Compared to vehicle, ^#^Compared to AIA control, ^@^Compared to indomethacin, ^$^Compared to Spirulina (200 mg/kg), n = 5–6.

The results of the present study indicated that the vehicle group, AIA control and Spirulina (400 mg/Kg) groups did not show mortalities during the course of the experiment. However, when comparing mortality in indomethacin and Spirulina (200 mg/Kg) groups with the vehicle group, Iindomethacin group showed 37.5% mortality while in Spirulina (200 mg/Kg) group it was 12.5% andthis was insignificantly different from the vehicle group (*P* = 0.2 and 1.0 respectively) (Fig 1a).

Clinical evaluation of the rats' ankles indicated that the AIA control group showed the top score (score 4) which was significantly greater than that observed in the vehicle group-. Treatment with Spirulina (200 or 400 mg/kg) produced a dose-dependent reduction in the clinical score of the ankles compared to AIA control group. Further,by using Kruskal Wallis Test to compare the median score of each group, the score obtained in rats treated with Spirulina (400 mg/kg) showed a statistically significant difference than other groups(*P*<0.001, Fig 1b).

Determination of serum TBARS level indicated an increase in AIA group compared to vehicle group. All the implemented agents reduced the serum TBARS level compared to AIA group. Treatment with Spirulina (400 mg/kg) significantly decreased serum TBARS level compared to indomethacin treated rats (Fig 1c). Compatible results were obtained with serum GSH level; the AIA group showed lower serum GSH compared to vehicle group. All the therapeutic regimens successfully increased serum GSH level in comparison to AIA control group (*P*<0.05, Fig 1d).

**Fig 2:**
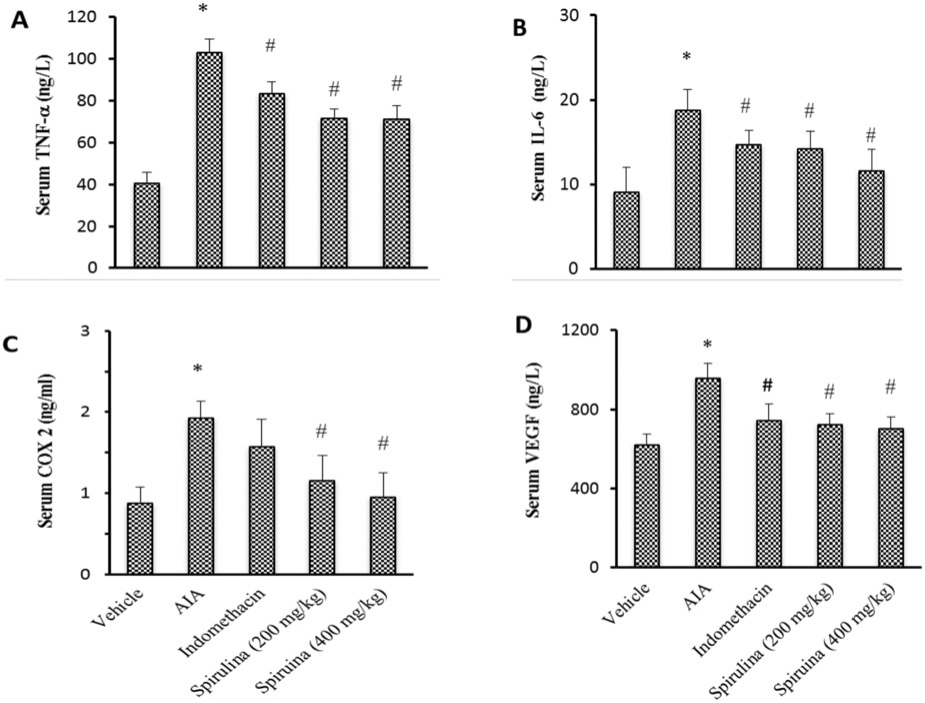
Effect of Spirulina (200 or 400 mg/kg) vs. indomethacin on serum level of TNF-α (A), IL-6 (B), COX-2 (C) and VEGF (D). Data are presented as mean ± SEM. *Compared to vehicle, ^#^Compared to CFA control, ^@^Compared to indomethacin, ^$^Compared to Spirulina (200 mg/kg), *n = 5–6*.

The current results demonstrated that serum level of TNF-α, IL-6, COX 2 and VEGF were greater in AIA control group in comparison to the vehicle group. Treatment with indomethacin lessened serum TNF-α, IL-6 and VEGF level without effect on serum COX 2. However Spirulina (200 or 400 mg/kg) reduced all these markers compared to AIA control group without difference with respect to indomethacin group (*P*<0.05, Fig 2).

**Fig 3:**
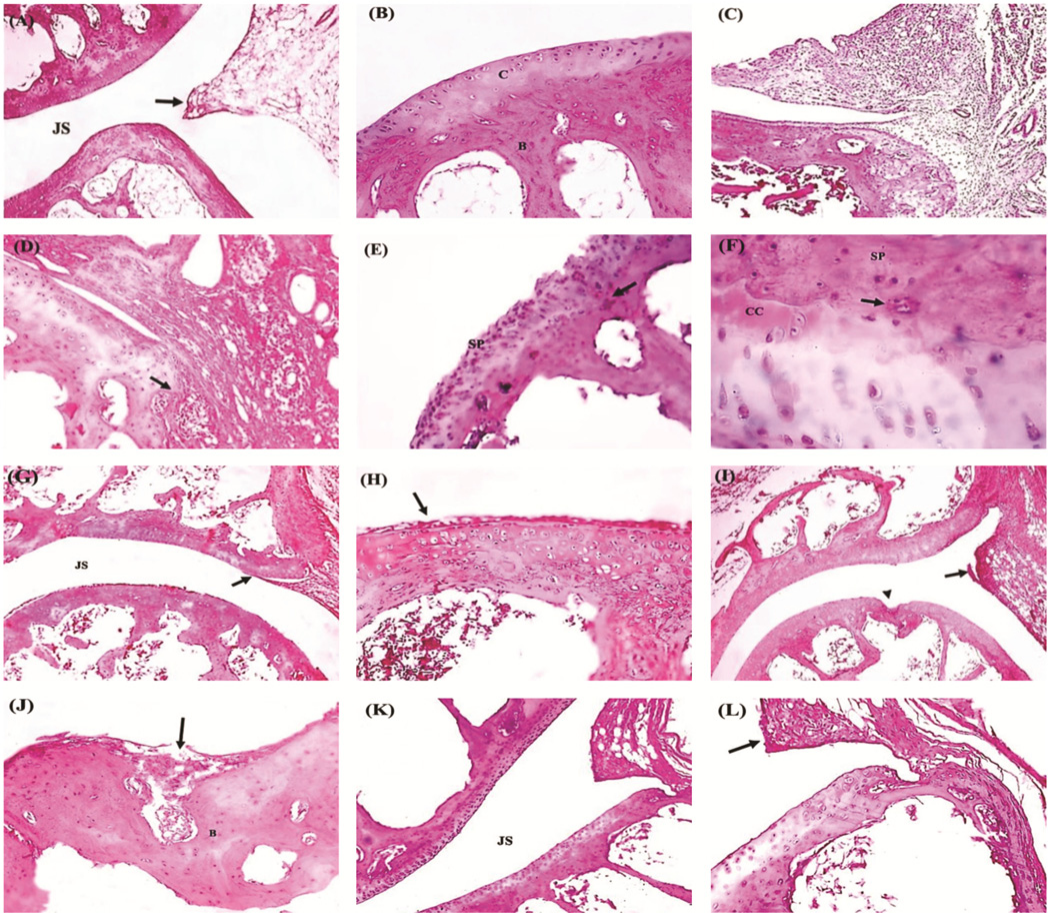
Photomicrographs for sections stained with hematoxylin & eosin. **(A)** Normal architecture of the joint showing the two articular cartilages separated by normal joint space (JS) with normal synovial membrane (arrow) and subsynovial connective tissue. **(B)** A higher magnification of the above section showing normal articular cartilage (C) and subchondral bone (B). **(C)** A section in the AIA group showing proliferated synovial membrane with grade 3 cellular infiltration (H&E x40). **(D)** A Higher magnification showing proliferating synovial membrane overlying and eroding the articular cartilage (arrow), denoting grade 3 synovial proliferation. **(E)** Complete destruction of the articular cartilage by synovial pannus (sp) (grade 3) which penetrates to the subchondral bone (arrow) in the control rats (AIA). **(F)** A higher magnification of the above section showing calcified articular cartilage (CC)—denoted by the darker hue of cartilage—eroded by overlying synovial pannus (sp). Notice the presence of mononuclear cell infiltration and multinuclear giant cell (arrow) in the synovial pannus. **(G)** A section in the Indomethacin group showing normal joint space (JS) but with grade 1 synovial proliferation (arrow). **(H)** A higher magnification showing few layers of synoviocytes (Arrow) overlying the articular cartilage denoting grade 1 synovial proliferation. **(I)** Spirulina (200 mg/kg) treated rats showed local destruction of articular cartilage (arrow head) and grade 2 synovial proliferation with the formation of synovial polyp (arrow). **(J)** Higher magnification showed grade 2 cartilage erosion (arrow) with destruction of the subchondral bone (B). **(K)** Spirulina (400 mg/kg) treated rats showed normal architecture of the joint with normal joint space (JC). **(L)** A higher magnification of the above section showing grade 1 synovial proliferation (arrow). Photomicrographs (A,C,G,H and K) at x 40; (B,D,E,J,I and L) at x 200 magnification; (F) at x 400 magnification.

Histopathological examination for H&E stained sections revealed that ankles from AIA group showed proliferating synovial membranes eroding the articular cartilage with grade 3 cellular infiltration, pannus formation and heavy mononuclear cell infiltration (Fig 3a). Joints from indomethacin treated rats showed decreased synovial proliferation and cell infiltration. On the other hand, ankles from Spirulina (200 mg/kg) treated rats showed grade 2 synovial proliferation and cartilage erosion, however, the high dose of Spirulina (400 mg/kg) showed fairly normal joint architecture and grade 1 synovial proliferation (Fig 3).

Comparison between the different groups illustrated that AIA group showed greater score for synovial proliferation, cell infiltration, cartilage erosion and pannus formation compared to vehicle group. Treatment with indomethacin or Spirulina (200 or 400 mg/kg) significantly reduced the total histologic scores compared to AIA group. Importantly, Spirulina (400 mg/kg) greatly suppressed the total scores compared to indomethacin group (Table 1).

**Table 1:** The scores of the histopathological changes in the different experimental groups.

*Histopathological changes*	C	I	S1	S2
***Synovial proliferation***				
Grade 0	—	—	—	2
Grade 1	—	3	2	2
Grade 2	—	1	2	—
Grade 3	4	—	—	—
*Total*	12	5	6	2
*Mean*	4	1.25	1.5	0.5
***Cellular infiltration***				
Grade 0	—	—	—	2
Grade 1	—	3	1	1
Grade 2	—	1	2	1
Grade 3	4	—	1	—
*Total*	12	5	7	3
*Mean*	4	1.25	1.75	0.75
***Cartilage erosion***				
*Grade 0*	—	1	—	3
*Grade 1*	—	3	2	1
*Grade 2*	2	—	2	—
*Grade 3*	2	—	—	—
*Total*	10	3	6	1
*Mean*	2.5	0.75	1.5	0.25
***Pannus formation***				
*Grade 0*	—	2	—	3
*Grade 1*	—	1	3	1
*Grade 2*	1	1	1	—
*Grade 3*	3	—	—	—
*Total*	11	3	5	1
*Mean*	2.75	0.75	1.25	0.25

**Fig 4:**
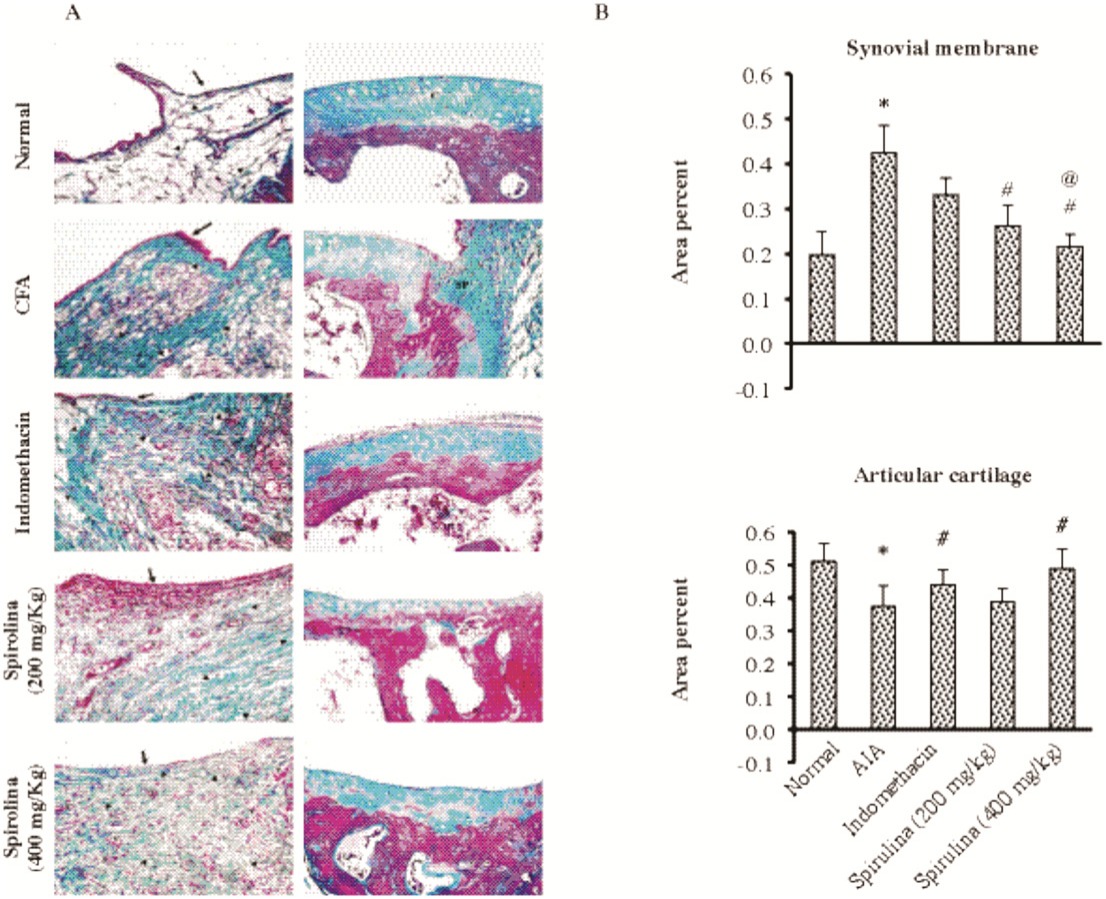
Histopathological picture for ankle specimens from the experimental groups stained with Masson’s trichrome stain (A). Section in the normal group is showing thin synovial membrane (arrow) with fine greenish color collagen fibers in the subsynovial connective tissue (arrow heads). The normal group also showed the greenish matrix of the articular cartilage (C). Section in the AIA control group showed proliferation of the synovial membrane (arrow), with heavy mononuclear cell infiltration and markedly thickened collagen fibers (arrow heads) in the subsynovial connective tissue compared to normal. Section in the AIA control group showing greenish synovial pannus (SP) invading and destroying the underlying articular cartilage which shows markedly altered stainability as it is reddish in color. Section in the Indomethacin group is showing thin synovial membrane (arrow) with heavy mononuclear cell infiltration and markedly thickened collagen fibers (arrow heads) in the subsynovial connective tissue compared to normal. Section in indomethacin group showing moderately altered stainability of the articular cartilage compared to normal. Spirulina (200 mg/kg) treated rats showed heavy mononuclear cell infiltration (arrow), increased vascularity (V) and moderately thickened collagen fibers (arrow heads) of the subsynovial connective tissue. Spirulina (200 mg/kg)-treated rats showed markedly altered stainability of the articular cartilage compared to the normal denoting poor quality collagen. Spirulina (400 mg/kg)-treated rats showed thin synovial membrane (arrow), with increased vascularity (V) but mild mononuclear cell infiltration and mildly thickened collagen fibers (arrow heads) of the subsynovial connective tissue. Spirulina (400 mg/kg)-treated rats showed moderately altered stainability of the articular cartilage (arrow head) compared to normal (Masson's Trichrome x200). **(B)** The area % in synovial membrane (top left panel) and the articular cartilage (bottom left panel). Data are presented as mean±SEM. *Compared to normal, ^#^Compared to AIA control, ^@^Compared to indomethacin, ^$^Compared to Spirulina (200 mg/kg).

Histopathologic examination for ankle sections stained with Masson’s trichrome demonstrated that AIA group showed high proliferation of synovial membrane accompanied by marked thickening of collagen fibers. Ankles from indomethacin-treated rats showed thin synovial membranes. Ankles from Spirulina-treated rats (200 mg/kg) showed moderately thickened collagen fibers (Fig 4a). The area % of the synovial membrane was greater whereas the area % for articular cartilage was smaller in AIA group compared to normal (Fig 4b). Spirulina (200 or 400 mg/kg) suppressed the area % of the synovial membrane compared to AIA control. Further, indomethacin and Spirulina (400 mg/kg) were able to ameliorate the area % for the articular cartilage.

## Discussion

Natural products have received considerable attention for the management of many degenerative diseases, including inflammation and RA [23,24]. Recently, *Spirulina platensis* is gaining more attention as a source of potential remedy for prevention and treatment of many diseases [25]. In the present study, the antioxidant and the immunomodulatory effects of *Spirulina platensis* as well as its anti-angiogenic effect against (CFA)-induced arthritis in rats model were tested. *S*. *platensis* obscured the development of arthritis in rats and inhibited the development of macroscopic and microscopic symptoms in CIA rats. Spirulina showed a higher survival rate than indomethacin-treated group. Although our study didn't investigate the reason for indomethacin-induced mortality, this is probably due to gastric and intestinal necrosis, erosions and ulceration associated with indomethacin treatment as reported by other work groups [26,27]. Moreover, it reduced the clinical score of RA in a dose dependent manner. In addition, Spirulina decreased serum levels of COX-2, TNF-α, IL-6, TBARS, VEGF and increased serum levels of GSH. Furthermore, it inhibited the development of histopathological lesions in CFA-induced arthritis in rat model.

Reactive oxygen species (ROS) are continuously produced inside the body as a result of exposure to many exogenous and endogenous chemicals [28, 29]. Normally, there is a balance between the ROS generated and the antioxidants present, as the ROS generated are neutralized by the endogenous antioxidants [29, 30]. Any imbalance between the generation and inactivation of these species leads to cellular function irregularities and different pathological conditions including RA [25, 29, 30].

Rheumatoid arthritis is always accompanied with lipid peroxidation and oxidative damage in human as well as in animal models [31, 32, 33]. In our rat model of CFA-induced arthritis, there was an elevation of serum TBARS and a decrease in serum GSH levels in RA non treated group. Spirulina triggered a significant protective effect by reducing TBARS content and increasing the level of GSH in a dose-depended manner. The effect of Spirulina is due to its antioxidant active components such as C-phycocyanins, β carotene, C-phycocyanins, vitamins and minerals. These results are in line with [29] who highlighted an antioxidant effect of Spirulina against deltamethrin-induced oxidative stress and toxicity in male rats. Moreover, our results are in agreement with those reported antioxidant effect of Spirulina against mercuric chloride-induced testis injuries and sperm quality deteriorations in rats [34].

Many researchers have shown that inflammatory cytokines have crucial roles in the pathogenesis of AIA, such TNF-α, IL-6, and COX- 2 [35]. In the present study, we observed a significant elevation in serum TNF-α, IL-6, COX- 2 concentration in AIA group in comparison to the control group. Treatment with indomethacin decreased serum TNF-α and IL-6 level without affecting the other inflammatory markers. However, Spirulina (200 and 400 mg/kg) reduced all previous markers compared to AIA group. *Spirulina platensis* and its active constituent C-phycocyanin reduced COX-2, IL-6 and TNF-α inducible nitric oxide synthase (iNOS) mRNA expression in microglial cells [36]. Moreover, it induced anti-inflammatory and antihyperalgesic activities in carrageenan-induced thermal hyperalgesia rat model through suppressing iNOS, COX-2 and PGE_2_ induction and attenuation of TNF-α formation and neutrophil infiltration into inflammatory sites [37].

Although the anti-angiogenic effect of NSAIDs is well documented in cancer [38, 39] their therapeutic anti-angiogenic potential in rheumatoid arthritis was recently explored [40].

Vascular endothelial growth factor is the best characterized growth factor expressed in RA. The expression of angiogenesis-regulating factors is changed in RA, so the inhibition of angiogenesis has been anticipated as a new therapeutic option in RA. Inhibition of angiogenesis has been shown to be useful in suppressing CFA-induced arthritis as anti-VEGF inhibited synovitis, indicated by a reduction in clinical score and paw swelling relative to control rats [41].

The current study demonstrated that serum level of VEGF was greater in AIA group in comparison to the control group. Treatment with Spirulina (200 and 400 mg/kg) reduced serum level of VEGF compared to CIA control group as well as indomethacin group, giving a new prospective aspects for Spirulina. Polysaccharide extract from *Spirulina platensis* inhibited VEGF as well as TNF-α in alkali burn-induced corneal inflammation and neovascularization [42].

## Conclusion

So far the results of the present study conclude that Spirulina is able to suppress the changes produced during adjuvant-induced arthritis which could be due to its immunomodulatory effect as well as its anti-angiogenic modality.
